# A MATLAB-based Instrument Control (MIC) package for fluorescence imaging

**DOI:** 10.21105/joss.07275

**Published:** 2025-01-28

**Authors:** Sajjad A. Khan, Sandeep Pallikkuth, David J. Schodt, Marjolein B. M. Meddens, Hanieh Mazloom-Farsibaf, Michael J. Wester, Sheng Liu, Ellyse Taylor, Mohamadreza Fazel, Farzin Farzam, Keith A. Lidke

**Affiliations:** 1 Department of Physics and Astronomy, University of New Mexico, Albuquerque, New Mexico, USA; 2 Nanoscience and Microsystems Engineering, University of New Mexico, Albuquerque, New Mexico, USA; 3 Department of Mathematics and Statistics, University of New Mexico, Albuquerque, New Mexico, USA

## Abstract

MATLAB Instrument Control (MIC) is a software package designed to facilitate data collection for custom-built microscopes. Utilizing object-oriented programming, MIC provides a class for each low-level instrument. These classes inherit from a common MIC abstract class, ensuring a uniform interface across different instruments. Key components such as lasers, stages, power meter and cameras are grouped under abstract subclasses, which standardize interfaces and simplify the development of control classes for new instruments. Both simple and complex systems can be built from these lower level tools. Since the interoperation is developed by the end user, the modes or sequence of operations can be flexibly designed with interactive or automated data collection and integrated analysis. MATLAB provides the ability to create GUIs and therefore MIC allows for both rapid prototyping and for building custom, high-level user interfaces that can be used for production instruments.

## Statement of need

Development of new microscopy methods often require integration of novel components and/or novel workflows between low-level hardware. Creating such software solutions for each new instrument or for prototyping can significantly slow down the development cycle. It is also often desirable to be able to quickly analyze incoming data during development. To address this critical need, we have developed the MATLAB Instrument Control (MIC) software package, freely available and specifically tailored for the customization and automation of complex, multi-component microscopy systems (Pallikkuth et al., 2018). MIC leverages MATLAB’s (MathWorks Inc.) robust environment for object-oriented programming, allowing users to control diverse instrumentation through a unified interface. Each instrument class within MIC inherits from a common abstract class, ensuring consistency while enabling flexibility to accommodate a variety of components like lasers, stages, and cameras. The source code for MIC has been archived to GitHub: https://github.com/LidkeLab/matlab-instrument-control.

MIC not only supports the development and control of new instruments but also integrates seamlessly with MATLAB’s comprehensive data and image analysis tools, as well as with Single Molecule Imaging Toolbox Extraordinaire (SMITE) (Schodt, Wester, et al., 2023) allowing researchers to process data in real-time during experiments. This capability is crucial for iterative testing and development in experimental setups. For example, the single molecule localization microscopy/single particle tracking (SMLM/SPT) analysis software suite SMITE was developed in sync with MIC, so acquiring data and performing analyses flow smoothly. Additionally, MIC is designed to be user-friendly for those familiar with MATLAB, offering customizable control classes, extensive export methods, functional tests, and graphical user interfaces for each instrument component.

**Figure-1:**
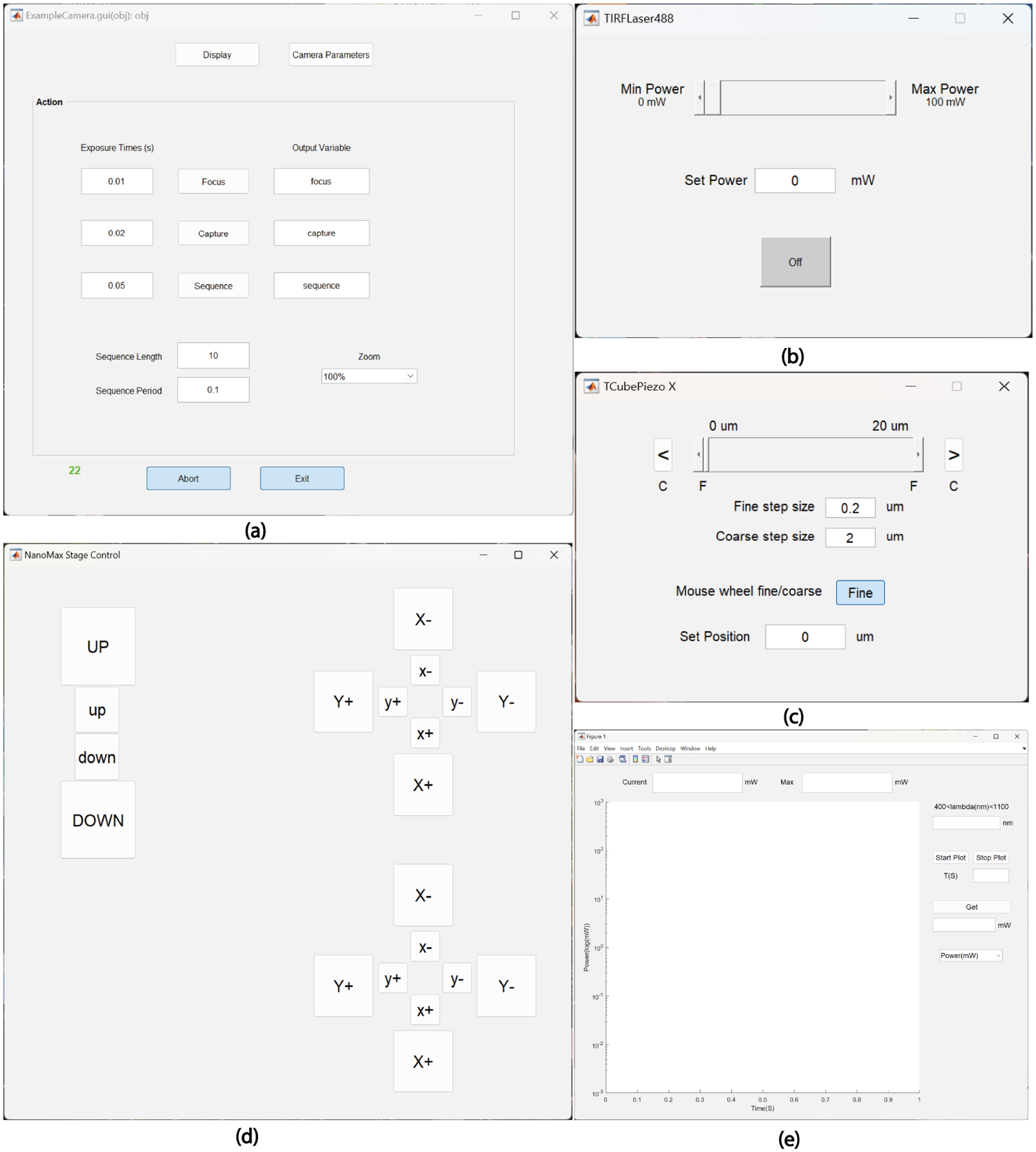
MIC Graphical User Interfaces (GUIs); (a) Camera control GUI (b) Light source (TIRF Laser488) GUI (c) Linear stage (TCubePiezo X) GUI (d) 3D stage (NanoMax Stage Control) GUI (e) Power meter GUI.

An example of MIC’s utility is demonstrated through a custom-built Sequential microscope available on Sequential SR Microscope (Schodt, Farzam, et al., 2023) specifically designed for direct STochastic Optical Reconstruction Microscopy (dSTORM) and DNA Point Accumulation for Imaging in Nanoscale Topography (DNA-PAINT) based super-resolution. Another usage of MIC is demonstrated through a class designed for Total Internal Reflection Fluorescence (TIRF) based super-resolution imaging TIRF SR Microscope (Fazel et al., 2022). Each of these classes includes an intuitive graphical user interface that manages multiple excitation lasers and camera settings, simplifying complex data collection tasks. Several MIC GUIs are presented in Figure-1.

MIC is designed to operate with Hierarchical Data Format (HDF5) files which efficiently store very large datasets. The HDF5 format is particularly useful for storing large datasets, as it allows for efficient data storage and retrieval. This is especially important for single molecule fluorescence imaging, where large amounts of data are generated during experiments. The HDF5 format is also supported by MATLAB, which makes it easy to import and analyze data stored in this format.

There are a few other software packages that allow users to control and synchronize multiple hardware components for microscopy applications. Notable examples include Micro-Manager (Edelstein et al., 2010), based in Java, PYME (the PYthon Microscopy Environment) (PYME Developer Community, 2020), based in the Python environment, and LSMAQ which is a lightweight and flexible laser scanning microscope acquisition software written in MATLAB. It supports National Instruments hardware for galvo-based scanning. Potential users of MIC are encouraged to compare and contrast MIC with these packages to assess what might be best for their particular development environment. Micro-Manager is a customizable platform for controlling microscopy systems, supporting a wide range of hardware devices, and is primarily built on Java. Micro-Manager comes with its own GUI. Micro-Manager can save files in three formats: separate image files, Image file stack (OME-Tiffs) and NDTiff. Micro-Manager may be a good choice for those who primarily use ImageJ/Fiji for image analysis. PYME is designed to facilitate image acquisition and data analysis in microscopy, with a focus on super-resolution techniques like PhotoActivated Localization Microscopy (PALM), STORM, and PAINT. It runs on multiple platforms, including Windows, Linux, and OSX. PYME comprises several key components: PYMEAcquire for microscope and camera control, PYMEVisualize for visualizing localization data sets, and PYMEImage for viewing and processing raster images. PYME is compatible with a variety of data formats, including its proprietary .pzf format as well as standard formats such as .tif. Additionally, PYME supports metadata in multiple formats, including .json, .md, and .xml.
